# Gene family assignment-free comparative genomics

**DOI:** 10.1186/1471-2105-13-S19-S3

**Published:** 2012-12-19

**Authors:** Daniel Doerr, Annelyse Thévenin, Jens Stoye

**Affiliations:** 1 Genome Informatics, Faculty of Technology, Center for Biotechnology (CeBiTec), Bielefeld University, Germany; 2 Institute for Bioinformatics, Center for Biotechnology (CeBiTec), Bielefeld University, Germany

## Abstract

**Background:**

The comparison of relative gene orders between two genomes offers deep insights into functional correlations of genes and the evolutionary relationships between the corresponding organisms. Methods for gene order analyses often require prior knowledge of homologies between all genes of the genomic dataset. Since such information is hard to obtain, it is common to predict homologous groups based on sequence similarity. These hypothetical groups of homologous genes are called *gene families*.

**Results:**

This manuscript promotes a new branch of gene order studies in which prior assignment of gene families is not required. As a case study, we present a new similarity measure between pairs of genomes that is related to the breakpoint distance. We propose an exact and a heuristic algorithm for its computation. We evaluate our methods on a dataset comprising 12 γ-proteobacteria from the literature.

**Conclusions:**

In evaluating our algorithms, we show that the exact algorithm is suitable for computations on small genomes. Moreover, the results of our heuristic are close to those of the exact algorithm. In general, we demonstrate that gene order studies can be improved by direct, gene family assignment-free comparisons.

## Background

In the field of comparative genomics, studying the relative order of genes in genomes is a popular practice to gain information about organisms and their relationships. This information ranges from transcription and functional linkage of genes such as correlated expression, the phylogeny of organisms, to detailed evolutionary dynamics of their genomes. Gene order methods are also incorporated in genome alignment strategies to identify regions that are subsequently used to anchor the alignment [1].

*Genes *are the atomic elements in gene order studies. Although no precise, formal definition is generally agreed upon, from the biological point of view a *gene *represents a specific *inheritable entity *in a particular *locus *on a chromosomal sequence in a particular organism. It often features a protein coding region. Nevertheless, the notion of a "*gene*" can also represent more fine-grained genetic structures such as protein domains or other functional elements of the genome.

**Gene families**. Many gene order studies hope for evolutionary relationships being resolved between all pairs of genes. Rested upon the biological concept of homology, such studies require information about orthology, paralogy and (potentially) xenology for each pair of genes in the dataset. This information is generally not given, hence it is common to cluster genes according to their sequence similarity. Sometimes such groups are called *gene families*, thus we will stick to this notion in the following.

Various databases exist, such as COG [2], eggNOG [3], Inparanoid [4], TreeFam [5], and OrthologID [6] (only to name a few) that offer gene family information. These databases can be divided into two groups: databases that primarily use sequence similarity to cluster genes into groups of co-orthologs; and tree-based databases offering reconstructed gene family trees [7].

The former group of databases provides usually more gene family data while covering a larger set of species. However, the contained information should always be taken with a pinch of salt: Even though high sequence similarity is a good indicator of homology, *per se *these gene families do not reflect an evolutionary relation. This is because they depend on arbitrary parameters of sequence comparison, similarity quantification, and clustering. Generally such parameters are user-controlled and influence the size and granularity of the computed gene families. Yet, the vast majority of these databases is uncurated or offers only a negligible amount of curated data.

Lacking a gene tree, within these gene families no differentiation can be made between in- and out-paralogs when comparing a specific pair of genomes. As is well-known, gene duplication and sub- or neofunctionalization occurs frequently in evolution. Hence the number of co-orthologous genes in a genome that are pooled into the same gene family grows the higher one ascends in the evolutionary tree. With increasing number of diverse genomes in the database, these gene families become less useful for gene order analyses, if only a close subset of taxa is of interest. The blemish of disregarding the evolutionary tree needed for truly resolving evolutionary relationships between genes of a given set of genomes is often covered by offering varying levels of granularity. This means that for some subtrees (but generally not for all) of the genomes in the database, gene families are recomputed with tighter parameters. Moreover, the computed sequence-based similarity estimates are rarely based on models of DNA evolution as these involve considerably more computational load. Subsequently differential evolutionary rates are disregarded, amplifying the dilemma of grouping genes based on sequence similarity: selecting too loose criteria in clustering genes to gene families may lead to the mistake that two genes are assigned to the same gene family while they are not homologous, whereas too strict criteria can split gene families although they should belong together [7].

Tree-based databases such as TreeFam and OrthologID may provide more accurate information desired for gene order studies. This is partly because the evolutionary relationships between genes in a gene family are considerd in more detail. Furthermore the species tree is taken into account while reconstructing the gene family trees. Also, tree-based databases tend to be more often manually curated than their sequence similarity based counterparts. In return, the provided gene family information is often sparse and covers not all genes of a genome. Moreover, such databases usually comprise only a handful of species. As a result, they are of limited use in gene order studies.

**Gene content variations**. Apart from model-free comparison or well-defined rearrangements in genomes, gene order studies can allow for additional biologically motivated operations of evolution. That is, genes can duplicate, emerge or become lost in the genome. Similarly, a gene family can grow or shrink, or new gene families can arise.

**Gene order studies**. Based on the concept of gene families, many gene order studies share a common data structure where chromosomes are represented as words drawn from a finite alphabet of gene families. The strength of this data structure lies in its simplicity; it allows to study the corresponding gene order problems in an abstract form composed of permutations or sequences over a set of characters. Another important advantage is the fact that homology is a binary and transitive relation. This led to the emergence of a multitude of efficient algorithms which solve gene order problems combinatorially.

In the following we will briefly review three different types of gene order studies. Dissimilarity measures such as the *breakpoint distance *[8] are used to calculate evolutionary distances between two or more genomes, without explicitly drawing on rearrangement operations. The breakpoint distance is defined by the number of unconserved adjacencies between characters of two genomes. For gene cluster detection, several competing models exist. One of them is based on the notion of *approximate common intervals *[9]. Thereby a gene cluster is defined as a set of maximal intervals, on two or more genomes, that share the same character set. Small differences between the set of characters constituting the gene cluster and the set of characters within the intervals are allowed. The number of tolerated differences as well as the minimal size of an interval is determined by a user-controlled parameter. Finally, a group of popular rearrangement models are based on the so-called *double-cut-and-join *(DCJ) operation [10,11]. By disrupting the genome on two different positions and rejoining the resulting ends, one aims to transform one genome into another by a minimal sequence of DCJ operations. This sequence is denoted *sorting scenario*.

**Limits of the gene family concept**. The concept of gene families comes with much benefit, but also has its detriments. On the one hand, gene family information can be gained with comparatively low effort by accessing various public databases or by direct computation. On the other hand, comparative studies based on uncurated gene families are hampered since data can be incorrect.

There are many reasons why the exclusive, binary membership relation between genes and gene families is disputable in itself. For one, most gene families are uncurated, hence it would be supporting in constitutive analyses to distinguish between weak and strong assumptions of homology between genes in supporting their membership to one or more gene families. Moreoever, the gene family concept disregards the facts that gene families may share conserved protein domains and that genes may fuse with others in the course of evolution.

In this paper we promote the idea that gene order studies can be performed without prior gene family assignment. We propose direct use of similarity values because such information not only allows to make more substantiated choices in resolving gene order in subsequent analyses, but can sometimes better reflect the biological reality. In support of our case, we present a new approach to calculate the number of conserved adjacencies, which is a similarity measure related to the breakpoint distance, without the use of gene families. Our method is based on a weighted bipartite graph, representing pairwise similarities between genes of two genomes. We show that this allows for stable adjacency analyses when similarities are calculated based on sequence similarity.

In the "Methods" section we will introduce the problem setting formally and devise an exact algorithm as well as a heuristic for its solution. In the "Experiments and Discussion" section we discuss the performance of our presented method on this dataset and compare results with former work. The manuscript closes with concluding remarks and future prospects in the "Conclusions" section.

## Methods

### Formal problem description

**Genome model**. Let G  be the universe of all genes, then a chromosome is defined as a sequence of genes (º, *g*_1_, *g*_2_, ..., *g*_*n*−1_,º), with $g_{i}\in G$ for all *i *= 1, ..., *n *− 1, flanked by *telomeric ends *represented by "º". Depending on the type of gene order study, chromosomes can be signed or unsigned. If signed, a gene *g *has a direction indicated by −*g *or +*g *(but it is common to omit the "+"), which represents the relative orientation of each gene along the chromosome. A chromosome can also be circular as it is often observed in bacteria; in this case, it does not exhibit telomeric ends, implying that the outermost genes adjoin. For the time being, let us assume that a *genome *is *unichromosomal *and *linear*, since the general case of our model can be easily inferred. The *size *of a genome *G *with *n *− 1 genes is |*G*| = *n*. In order to refer to the *i*th gene of *G*, we use the notation *G*[*i*]. Further, let σ:G×G→[0,1] be a *normalized similarity measure *between all pairs of genes.

**Figure 1:**
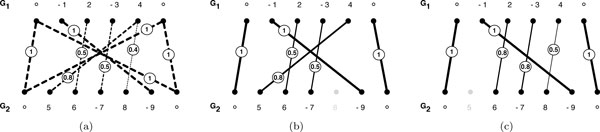
**Bipartite graph and matching**. **(a) **The ordered weighted bipartite graph represents similarity relationships between genes of two genomes. **(b) **and **(c) **represent solutions to Problem 1 with *α *→ 0 and *α *= 1 respectively.

**Graph representation**. Given two genomes *G*_1_, *G*_2 _of lengths *n*_1 _= |*G*_1_| and *n*_2 _= |*G*_2_|, respectively. We define an *ordered weighted bipartite graph B *= (*G*_1_, *G*_2_, *E*) over both genomes in which the order is given by the chromosomal order of genes (see example in Figure 1(a)). For 0 <*i *<*n*_1_, 0 <*k *<*n*_2_, a pair of genes, one from *G*_1 _and one from *G*_2_, is connected by an edge *e_ik _*:= (*G*_1_[*i*], *G*_2_[*k*]) ∈*E *with edge weight *w*(*e_ik_*):= σ(*G*_1_[*i*], *G*_2_[*k*]) if and only if σ(*G*_1_[*i*], *G*_2_[*k*]) > 0. Telomeres are always connected with edges of weight 1: $w(e_{00})=w(e_{0n_{2}})=w(e_{n_{1}0})=w(e_{n_{1}n_{2}})=1$, as depicted in our example in Figure 1(a). We call a gene *g_i _*∈ *G_x_*, *x *∈ {1, 2} *unconnected *if there exists no gene *g_k _*∈ *G_y_*, *y *∈ ({1,2}\{*x*}) such that σ(*g_i_,g_k_*) > 0.

Unconnected genes are omitted from the chromosomal sequences. The remaining genes form connected components of size two or larger. Let C  denote the set of all such connected components of *B*, then for some C∈C and *x *∈ {1,2}, *C_x _*denotes the set of all genes of *C *that are part of *G_x_*. Given *B*, we will be interested in finding a set of disjoint edges. Such a set, denoted by M , is known as matching.

**Matchings**. Let us assume for now that a matching M  between *G*_1 _and *G*_2 _is given. #edg(M ) denotes the number of edges in M . We call a gene *saturated *if it is incident to an edge of the matching. A pair of genes (*G_x_*[*i*], *G_x_*[*j*]), with *x *∈ {1,2} and 0 ≤ *i *<*j *≤ *n_x_*, is a *consecutive *pair if no saturated gene lies between them.

Recall that genes have directions; the orientation of a gene *g *is determined by the following function:

$$sgn\left(g\right)=\left\{\begin{array}{cc} 1 & \text{if}\;g>0\\ -1 & \text{if}\;g<0\\ 0 & \text{if}\;g\;\text{is}\;\text{a}\;\text{telomere} \end{array}\right)$$

Two consecutive pairs of genes (*G*_1_[*i*], *G*_1_[*j*]) and (*G*_2_[*k*], *G*_2_[*ℓ*]), with 0 ≤ *i *<*j *≤ *n*_1 _and 0 ≤ *k*, *ℓ *≤ *n*_2_, form a *conserved adjacency *if the corresponding edges *e_ik_*, *e_jℓ _*are part of M  and:

1. for *k *<*ℓ*, *sgn*(*G*_1_[*i*]) = *sgn*(*G*_2_[*k*]) and *sgn*(*G*_1_[*j*]) = *sgn*(*G*_2_[*ℓ*]) or

2. for *k *>*ℓ*, *sgn*(*G*_1_[*i*]) ≠ *sgn*(*G*_2_[*k*]) and *sgn*(*G*_1_[*j*]) ≠ *sgn*(*G*_2_[*ℓ*]).

For example, in Figure 1(b) the consecutive gene pairs (2, −3) and (6, −7) represent a conserved adjacency. Telomeres located at the first and last position of the chromosomes are "unsigned" and thus can be used to form adjacencies in both directions. We denote the sum of all conserved adjacencies in a matching M  by #adj(M ).

Among all possible matchings between *G*_1 _and *G*_2_, we search the biologically most relevant. A well-known matching is the *maximal weighted matching*, which maximizes the sum of weights of disjoint edges of a bipartite graph. In our example, Figure 1(b) represents a maximal weighted matching. This kind of matching can be motivated from a biological point of view: The higher the sequence similarity between two genes, the more likely they are homologs. Yet, if we want to construct a biologically meaningful matching, we must not only consider edge weights, but also the ability of two edges forming a conserved adjacency in the final matching. We somehow want to maximize for the number of conserved adjacencies in the final matching, because we observe from biological data that rearrangements of genes in genomes occur parsimoniously. However, we want to prevent that conserved adjacencies incorporating low-weight edge pairs are formed if the corresponding genes are incident to higher-weight edges (see Figure 1(c)). Consequently we propose the following scoring scheme for conserved adjacencies:

(1) $$s(i,j,k,l)=\{\begin{array}{r} w(e_{ik})\cdot w(e_{jl})\\ 0 \end{array}\begin{array}{l} \;\text{if}\;(G_{1}[i],G_{1}[j])\;\text{and}\;(G_{2}[k],G_{2}[\ell ])\;\text{form }\;\text{a}\;\text{conserved}\;\text{adjacency}\\ \;\text{otherwise} \end{array}$$

In our matching we want to promote conserved adjacencies but also edges: Because in the presented approach, connected components are larger than gene families, we aim to match more than one pair per connected component, even in the case they do not exhibit adjacencies. Hence we quantify the quality of a matching M  according to the following functions, where *i, j *indicate indices in genome *G*_1_; *k*, *ℓ *in *G*_2_:

(2) $$adj\left(M\right)=\underset{\begin{array}{c} 0\leq i<j\leq \left|G_{1}\right|,\\ 0\leq k,l\leq \left|G_{2}\right| \end{array}}{\sum }s\left(i,j,k,l\right)$$

(3) edgM = ∑e∈M we2

Notice that the edge weights in the sum of the Equation 3 are squared to match the dimension of Equation 2. Optimizing a matching with respect to edg(M) will result in a maximal weighted matching in the graph model we introduced above. As our overall objective function we propose a linear combination between Equations 2 and 3. We allow the user to balance between those two quantities by a parameter *α*. Moreover it is reasonable to add the constraint that at least one edge per connected component of the bipartite graph between *G*_1 _and *G*_2 _must be contained in the matching; The matching obtained is an *intermediate matching*.

**Problem 1 (Family-free(FF)-Adjacencies) ***Given two genomes G*_1 _*and G*_2_*, a normalized similarity measure σ, and some α ∈ *]0, 1]*, find a matching *M *in B = (G*_1_*, G*_2_*,E) such that at least one edge per connected component of B is contained in *M *and the following formula is maximized:*

(4) F αM =α⋅adjM +1-α⋅edgM .

Problem **FF-Adjacencies **can be reduced to two problems that were addressed already by Tang and Moret [12] and Angibaud *et al. *[13]. Therefore, let us consider equivalent conditions that prevail if gene families are given: In the bipartite graph *B *= (*G*_1_, *G*_2_,*E*) between two genomes *G*_1 _and *G*_2 _all edges have edge weight 1 and all connected components are *cliques*. Then finding a solution to Problem **FF-Adjacencies **with *α *= 1 is equivalent to finding a matching that maximizes the number of adjacencies between two genomes with duplicate genes under the intermediate model [13]. If *α *comes close enough to 0, we will obtain a maximum matching, yet maximizing the number of adjacencies [12]. The case where family conditions are met also reveals the difference between an arbitrary maximum matching and the maximum matching found by solving Problem **FF-Adjacencies **for *α *→ 0.

The reduced problems presented above being already NP-hard, the problem **FF-Adjacencies **is NP-hard as well. In the next two subsections we propose first an exact algorithm, **FFAdj-Int**, to solve Problem **FF-Adjacencies **and then a fast heuristic approach.

### Exact algorithm

Our algorithm **FFAdj-Int **solving Problem **FF-Adjacencies **is based on previous work in [13]. The idea is to translate the problem into a 0-1 linear program. That means we define a set of constraints (linear inequations) whose variables are booleans and an objective function (maximization or minimization of a linear formula). Then, we use a solver to assign a value for each variable such that the constraints are verified and the objective is optimized.

**Figure 2:**
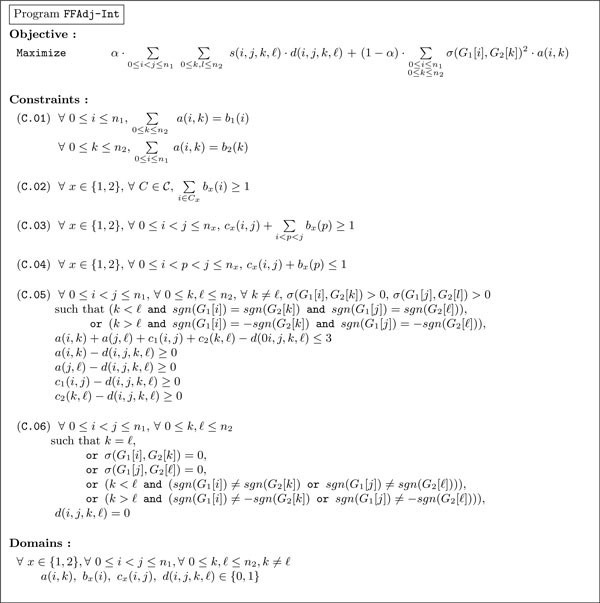
**Program FFAdj-Int**. Program **FFAdj-Int **for finding an intermediate matching that maximizes the objective F *_α _*(*α *∈ ]0, 1]) between two genomes *G*_1 _and *G*_2_.

The program **FFAdj-Int **considers two linear genomes *G*_1 _and *G*_2 _of respective lengths *n*_1 _and *n*_2_, a number *α *∈ ]0, 1], and a function *σ *: G  × G  → [0,1]. The objective, the variables and the constraints are defined in Figure 2 and are briefly discussed hereafter.

Variables:

• Variables *a*(*i*, *k*), 0 ≤ *i *≤ *n*_1 _and 0 ≤ *k *≤ *n*_2_, define a *matching *M : *a_i,k _*= 1 if and only if the gene at position *i *in *G*_1 _is matched with the gene at position *k *in *G*_2 _in M , *i.e*. *e_ik _*∈ M .

• Variables *b_x_*(*i*), *x *∈ {1, 2} and 0 ≤ *i *≤ *n_x_*, represent the genes *saturated *by M : *b_x_*(*i*) = 1 if and only if the gene at position *i *in *G_x _*is saturated by the matching M . Clearly, Σ_0 ≤ *i *≤ *n*1 _*b*_1_(*i*) = Σ_0 ≤ *k *≤ *n*2_* b*_2_(*k*), and this is precisely the size of the matching M .

• Variables *c_x _*(*i, j*), *x *∈ {1, 2} and 0 ≤ *i *<*j *≤ *n_x_*, represent *consecutive pairs *according to the matching M : *c_x_*(*i*, *j*) = 1 if and only if the genes at positions *i*, *j *in *G_x _*are saturated by M  and no gene at position *p*, *i*<*p*<*j*, is saturated by M .

• Variables *d*(*i*, *j*, *k*, *e*), 0 ≤ *i *<*j *≤ *n*_1_, 0 ≤ *k*, *ℓ *≤ *n*_2_, represent conserved adjacencies according to the matching M : *d*(*i*, *j*, *k*, *e*) = 1 if and only if *s*(*i*, *j*, *k*, *ℓ*) > 0.

Because the matching is possible only between similar genes, the variables *a*(*i*, *k*) and *d*(*i*, *j*, *k*, *ℓ*) are not defined whenever *σ*(*G*_1_[*i*], *G*_2_[*k*]) = 0. Similarly, the variables *d*(*i*, *j*, *k*, *ℓ*) are not defined if *σ*(*G*_1_[*j*],*G*_2_[*ℓ*]) = 0.

Objective:

The goal of **FFAdj-Int **is to find a matching M  between the two considered genomes that maximizes the formula F *_α _*(*α *∈ ]0, 1]). Hence, the objective of **FFAdj-Int **reduces to maximizing the sum of all variables *d*(*i*, *j*, *k*, *ℓ*) multiplied by *α *· *s*(*i*, *j*, *k*, *ℓ*), plus the sum of all variables *a*(*i*, *k*) multiplied by (1 −*α*) · *σ*(*i*, *k*)^2 ^.

Constraints:

Assume *x *∈ {1, 2}, 0 ≤ *i *<*j *≤ *n*_1 _and 0 ≤ *k*, *ℓ *≤ *n*_2_.

• Constraints in (**C.01**) ensure that each gene of *G*_1 _and of *G*_2 _is saturated at most once, *i.e*. *b*_1_(*i*) = 1 (resp. *b*_2_(*k*) = 1) if and only if there exists a unique *k *(resp. *i*) such that *a*(*i*, *k*) = 1, *i.e*. *e_ik _*∈ M .

• Constraints in (**C.02**) ensure that the matching M  is an intermediate matching, we want for each component at least one edge in the matching M . For each component C∈C, the sum of the variables *b_x_*(*i*) for *i *∈ *C_x _*must be greater than or equal to 1.

• Constraints in (**C.03**) and (**C.04**) express the definition of consecutive pairs, thus fixing the values of the variables *c_x_*. The variable *c_x_*(*i*, *j*) (0 ≤ *i *<*j *≤ *n_x_*) is equal to 1 if and only if there exists no *p *such that *I *<*p *<*j *and *b_x_*(*p*) = 1. It is worth noticing that the constraints do not force the variables *c_x_*(*i*, *j*) to have exactly the values we intuitively wish according to the above mentioned interpretation. Here, we accept that *c_x_*(*i*, *j*) = 1 even if the gene at position *i *or *j *is *not *saturated. However, this will pose no problem in the sequel.

• Constraints in (**C.05**) and (**C.06**) define variables *d*. Knowing the variables *d*(*i*, *j*, *k*, *ℓ*) are defined only if *σ*(*i*, *k*) > 0 and *σ*(*j*, *ℓ*) > 0, constraints (**C.05**) and (**C.06**) ensure that we have *d*(*i*, *j*, *k*, *ℓ*) = 1 if and only if all variables *a*(*i*, *k*), *a*(*j*, *e*), *c*_1_(*i*, *j*) and *c*_2_(*k*, *e*) are equal to 1 and the signs and the order of *G*_1_[*i*], *G*_1_[*j*], *G*_2_[*k*] and *G*_2_[*ℓ*] are consistent with the definition of conserved adjacencies.

The program **FFAdj-Int **has *O*((*n*_1_*n*_2_)^2^) constraints and *O*((*n*_1_*n*_2_)^2^) variables, which could result in a time-consuming computation.

So far we have used only one simple rule in order to reduce the space complexity: By the definition of the intermediate model, for all components with only two genes, *G*_1_[*i*] and *G*_2_[*k*], the edge *e_ik _*is in M . By the constraints (**C.01**) and (**C.03**), we already enforce that the variables *a*(*i*, *k*), *b*_1_(*i*) and *b*_2_(*k*) are equal to 1. The rule is based on the fact that there is no possible consecutivity in M  between *G*_1_[*s*] and *G*_1_[*t*] (resp. *G*_2_[*s*] and *G*_2_[*t*]) such that 0 ≤ *s *<* i *<* t *≤ *n*_1 _(resp. 0 ≤ *s *<*k *<*t *≤ *n*_2_), *i.e*. *c*_1_(*s*, *t*) (resp. *c*_2_(*s*, *t*)) is equal to 0. The corresponding variables *d*(*s*, *t*, ., .) (resp. *d*(., ., *s*, *t*) and *d*(., ., *t*, *s*)) are also equal to 0.

### Heuristic

Because of the combinatorial explosion, **FFAdj-Int **does not solve Problem **FF-Adjacencies **for all pairs of complete, larger genomes. But, we will see in the "Experiments and discussion" section that **FFAdj-Int **allows to obtain enough results to evaluate our heuristic presented in this section. It is based on similar ideas as the heuristic **IILCS **in [13]. **IILCS **allows to compute the number of adjacencies between two genomes when gene families are known, under three models: exemplar (only one match by gene family), intermediate, and maximum. **IILCS **resolves our Problem **FF-Adjacencies **in the particular case where *α *= 1 and each component represents a gene family, *i.e*. each component is a clique where the weight of each edge is 1.

The heuristic IILCS is a greedy algorithm based on the notion of *LCS*, Longest Common Substring: Given two genomes *G*_1 _and *G*_2_, an *LCS *is a longest string *S *such that *S *is a (consecutive) substring in *G*_1 _and *G*_2_, up to a complete reversal (opposite sign and reverse order). The idea is to match, at each iteration, all the genes that are in an *LCS*. If there are several *LCSs*, one is chosen arbitrarily. At each iteration, not only we match an *LCS*, but we also remove each unmatched gene from the genome, for which there is no unmatched gene of same component in the other genome. The process (determination of LCS, match and deletion of genes) is iterated until a satisfying matching is obtained. Under the intermediate model, the iteration is stopped when there is at least one edge in M  for each component.

For the problem **FF-Adjacencies**,we update the **IILCS **heuristic by three modifications. The goal of the first change is to take into account our objective in the choice of common substrings. In each iteration we match the common substring that maximizes locally F *_α _*(*α *∈ ]0,1]), i.e. the sum of weights of adjacencies and edges. We call this common substring a *Maximum Common Substring *(MCS). The second modification is an improvement that may also be applied to the original **IILCS **heuristic: After the deletion of an unsaturated gene *g*_1_, such that there is no unmatched gene *g*_2 _with *σ*(*g*_1_,*g*_2_) > 0, we attempt to increase the size of each previously matched MCS by extending it on both extremities. The next and the last change is related to the model. We have two options to increase our objective. The first one is to stop the iteration only when we have at least one edge per component and when the size of the MCS of the current iteration is below 2. In the case of the gene family constraints, this criterion improves also the results of **IILCS**. The second possibility is to stop the iteration only when there is no more edge between unmatched genes. In comparison to the first possibility, we increase our objective F *_α _*(*α *∈ ]0,1]) only if *α *≠ 1, so not in the context of **IILCS**. We choose this second possibility because the objective is bigger, but it is important to understand that then we also increase the number of breakpoints. We call this heuristic **FFAdj-MCS**.

## Experiments and Discussion

### Data

To evaluate our algorithms, we chose 12 γ-proteobacteria genomes from the dataset of Lerat *et al. *that was also used in previous work and which is to some degree considered as a standard reference dataset in comparative genomics [13-15]. The suggested phylogeny has been confirmed in later studies e.g. Williams *et al. *[16]. The genomic data including gene annotations have been obtained from NCBI under the accession numbers listed in Table 1.

**Table 1:** Genomic dataset.

Species/strain name	Short name	Accession No.	Size (bp)	#Genes
*Buchnera aphidicola *APS	BAPHI	NC_002528	640681	564
*Escherichia coli *K12	ECOLI	NC_000913	4639675	4320
*Haemophilus influenzae *Rd	HAEIN	NC_000907	1830138	1657
*Pseudomonas aeruginosa *PA01	PAERU	NC_002516	6264404	5571
*Pasteurella multocida *Pm70	PMULT	NC_002663	2257487	2012
*Salmonella typhimurium *LT2	SALTY	NC_003197	4857432	4423
*Wigglesworthia glossinidia brevipalpis *	WGLOS	NC_004344	697724	611
*Xanthomonas axonopodis pv. citri *306	XAXON	NC_003919	5175554	4312
*Xanthomonas campestris *	XCAMP	NC_003902	5076188	4179
*Xylella fastidiosa *9a5c	XFAST	NC_002488	2679306	2766
*Yersinia pestis *CO_92	YPEST-CO92	NC_003143	4653728	3885
*Yersinia pestis *KIM5 P12	YPEST-KIM	NC_004088	4600755	4048

The genomic dataset of our analysis comprises 12 *γ*-proteobacteria from Lerat *et al. *[14].

All genomes comprise a single, circular chromosome. In support of simplified code but at the expense of accuracy, our implemented algorithms do not allow a chromosome to be circular, even though this is permitted by our presented model. However, the maximal error made by this inaccuracy in comparing two genomes is at most one adjacency, which is negligible in our analysis. The genomes were linearized in the order inherent to the NCBI data, and telomeres were added at the beginning and at the end of the resulting chromosomal sequences.

Pairwise normalized similarities were obtained using the *relative reciprocal BLAST score *(RRBS) [17]. Genes were compared on the basis of their encoding protein sequence using BLASTP with an e-value threshold of 0.1, disabled query sequence filtering, and disabled composition-based score adjustments. All computations were performed on a computer system with 32 gigabytes of main memory.

### Exact Algorithm vs Heuristic

**Figure 3:**
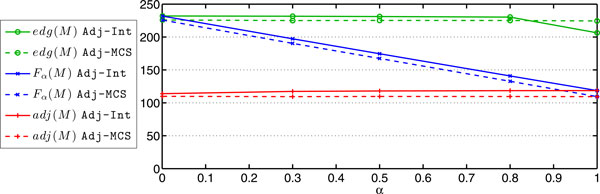
**Solving FF-Adjacencies for various values of *α***. Mean values of F *_α_*(M ), *adj*(M ) and *edg*(M ) plotted as a function of *α *for 40 genomes based on results of **FFAdj-Int **(solid lines) and **FFAdj-MCS **(dashed lines).

Using the CPLEX solver we ran the 0-1 linear programs obtained by **FFAdj-Int **for 66 pairs of genomes with varying values of *α *(*α *∈ {0.001, 0.3, 0.5, 0.8, 1}). A detailed table of results is enclosed in Additional file 1. In some cases, in particular larger and close genomes, we were not able to obtain results due to the lack of sufficient memory; We obtained results for 43 (resp. 63, 61, 54 and 48) pairs of genomes for *α *= 0.001 (resp. 0.3, 0.5, 0.8 and 1). For 40 pairs of genomes we could solve the 0-1 linear program for all values of *α*. These results are summarized in Figure 3, showing mean values of F *_α_*(M ), *adj*(M ), and *edg*(M ) plotted as a function of *α*. The same plot also includes results of our heuristic showing similar trends. Both, *adj*(M ), and *edg*(M ) show little change while varying *α*. This indicates that the set of high-scoring adjacencies and high-weight edges, that contribute the most, are largely shared among the matchings with different *α*. The abrupt drop in the mean value of *edg*(M ) for *α *= 1 results from the fact that for this value the second term of Equation 4 drops out. Consequently, single pairs (i.e. those that do not share conserved adjacencies) of matched genes (*G*_1_[*i*], *G*_2_[*k*]) are removed from the genomes during the resolution of the linear program. On the other side, because the heuristic iteratively constructs a matching until full saturation, the value for *edg*(M ) for *α *= 1 remains high.

In our evaluation of **FFAdj-MCS**, we demonstrate that it represents a feasible heuristic for Problem **FF-Adjacencies**. In Table 2 the relative deviation of the heuristic results from the solutions of **FFAdj-Int **are listed. In the worst case, where *α *= 1, the heuristic deviates in the objective by less than 10%. Due to its greedy nature, in all cases but one the size of the matching is larger than in the optimal solution. In order to evaluate the gain of the family-free analysis, we compare the results of **FFAdj-Int **against those of **Adjacencies-Intermediate-Matching **[13]. The linear program **Adjacencies-Intermediate-Matching **maximizes the number of adjacencies under the intermediate model between two genomes with gene family constraints. To compare the number of adjacencies (a common measure of these two programs) correctly, we must take into account two facts. First, the number of genes of the studied genomes differ. In [13], the authors used gene annotations and gene families that are reported in [15] whereas in our current study we employed gene annotations from NCBI. Nevertheless, the difference in number of genes is on average 0.02% per genome. Secondly, the genes for **Adjacencies-Intermediate-Matching **are unsigned, which artificially increases the number of adjacencies. We observed many more adjacencies in the results of **FFAdj-Int ** and of **FFAdj-MCS** than in **Adjacencies-Intermediate-Matching**. Furthermore, the matching produced by both **FFAdj-Int ** and **FFAdj-MCS **is on average larger than in Adjacencies-Intermediate-Matching.

**Table 2:** Exact algorithm vs heuristic.

	Relative deviation				RF distance	
Α	F **_α_(**M **)**	#adj	#edg	#exact results	exact	heuristic
0.001	-2.67%	2.83%	-0.23%	43	2	2
0.3	-3.47%	0.90%	0.31%	63	2	2
0.5	-4.26%	-1.03%	0.84%	61	2	2
0.8	-6.34%	-1.71%	1.14%	54	4	2
1	-8.41%	-2.39%	17.7%	48	6	2

Comparison of **FFAdj-Int **and **FFAdj-MCS**. The left side of the table shows the *relative deviation *in the objective function F *_α_*(M ), the number of adjacencies (**#adj**), and the size of the resulting matching (**#edg**), of heuristic results from the exact solutions under varying values of *α*. On the right, the RF distances between the true tree and trees based on exact and heuristic results are listed.

### Evaluating phylogenies

A good indicator for accuracy of a genome-based distance measure is the quality of the phylogenetic tree based on its drawn distances.

The distance measure that we used in this analysis resembles the breakpoint distance normalized by the size of the matching. For a given matching M  of a size #**edg**(M ) and a given number of adjacencies #**ad**j(M ), the normalized number of breakpoints is (#**edg**(M ) − #**adj**(M ) − 1)/#**edg**(M ). Now, since the objective of Problem **FF-Adjacencies **does not maximize adjacencies but rather a linear combination of *adj*(M ) and *edg*(M ), we define a distance measure based thereon:

$$\Delta \left(M\right)=\frac{edg\left(M\right)-adj\left(M\right)-1}{edg\left(M\right)}$$

**Figure 4:**
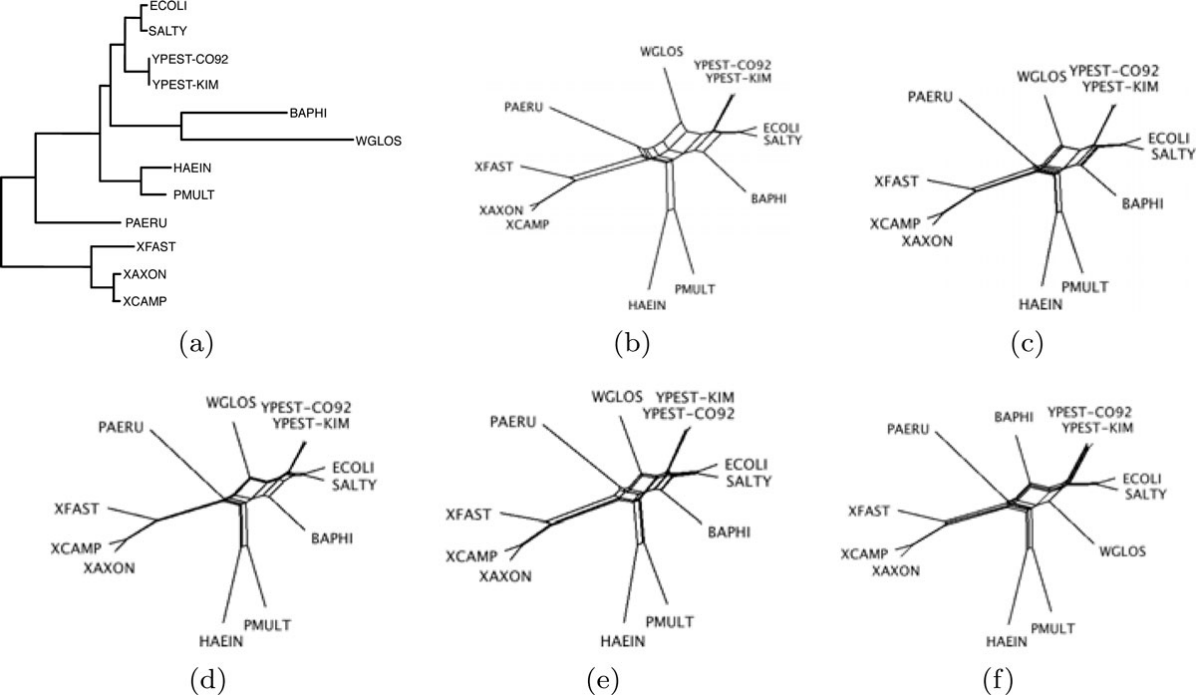
**Phylogenies**. **(a) **True phylogeny obtained from [14]. NeighborNet representations of phylogenies based on distances obtained by **(b) Adjacencies-Intermediate-Matching **[13], **(c) **running **FFAdj-Int **with *α *= 0.001, **(d) ***α *= 0.5, **(e) ***α *= 0.8, and **(f) **running **FFAdj-MCS **with *α *= 1.

In our evaluation we applied the well-known *Neighbor Joining Method *(NJ) [18] for inferring phylogenetic trees. Subsequently we compared these to the tree proposed by Lerat *et al. *[14] that we assume to represent the true phylogeny. Thereby we used the *Robinson Foulds topological distance *(RF distance) [19] to evaluate our inferred phylogenetic trees. The results are shown on the right side of Table 2. For the majority of all cases we were able to reconstruct the tree correctly up to a single internal edge, causing an RF distance of 2 to the original tree. This internal edge connects the two organisms *Buchnera aphidicola *(BAPHI) and *Salmonella typhimurium *(SALTY) with the rest of the tree (Figure 4(a)). This branch is known to be particularly hard to reconstruct since the two organisms diverged far from each other, resulting in two long external edges in the tree. We also reconstructed the phylogeny based on gene families using the measures that were proposed in [13] and obtained an RF distance of 2 to the true tree under the intermediate model and an RF distance of 4 under the maximum matching model, featuring the same aberrancy. These results suggest that gene family information is not relevant in reconstructing the phylogeny of Lerat *et al*.'s tree. Yet, the increasing deviation from the true tree for the results of **FFAdj-Int **when *α *tends to 1 indicates that for genome comparison, the maximization of adjacencies is not enough. The fact that the heuristic outperforms the exact algorithm for *α *= 1 in terms of RF distance to the true tree confirms the importance of maximizing *edg*(M ) as well. We recall that **FFAdj-MCS **iterates until complete saturation is obtained, which increases *edg*(M ), even when *α *= 1.

Often, one cannot judge the tree-additivity of the underlying distances by investigating the fully resolved Neighbor Joining tree. Thus, in Figure 4 we provide a NeighborNet [20] representation of some of our obtained phylogenies. In the plots the internal edges that are hard to reconstruct are directly exposed, showing network-like rather than tree-like structures, in particular for the tree obtained from [13]. To conduct these phylogenetic analyses, we used the software packages PHYLIP [21] and SPLITSTREE [22].

## Conclusions

In this work, we introduced the concept of comparative genomics by direct analysis of gene similarities without prior assignment of gene families. To illustrate this approach, we resorted specifically to one problem of gene order comparison: Finding a matching that identifies similarities between two genomes by maximizing conserved adjacencies and similarities for each pair of genes simultaneously. This problem is NP-hard. We propose to resolve it by an exact algorithm (efficient for small genomes) and a good heuristic. In our experiments on 12 γ-proteobacterial genomes, we observed that the omission of gene families allowed for an increase in the number of adjacencies as well as the size of the matching while the resulting distances gain higher precision in reconstructing phylogenies.

**Future work**. This study is a preliminary work in a new field of comparative genomics wherein the assignment of gene family is unnecessary. Many studies can be explored. With regard to the specific problem studied here, our exact algorithm can be improved by rules which reduce the required main memory. Moreover, we believe that a hybrid heuristic - starting a pre-matching using the iterative heuristic until the size of the MCS is less than a parameter *k*, then finishing the matching with our exact algorithm - can allow to find near-exact results for even larger genomes. On the other side, a deep study of the measure σ can increase the quality of the comparison; comparing genes by sequence similarity is only one of many methods that can be applied.

From a more general point of view, this study shows that it is conceivable to extend the direct analysis approach to other types of gene order studies such as the computation of DCJ distances or gene cluster prediction.

## Competing interests

The authors declare that they have no competing interests.

## Authors' contributions

All authors participated in discussing, formulating, and conducting the research. Also, all authors contributed to the writing and editing of the manuscript and read and approved the final manuscript.

## Supplementary Material

Additional file 1 Measured adjacencies between 12 γ-proteobacterial genomes. Values obtained from **FFAdj-Int **and **FFAdj-MCS**. **#adj **denotes the number of conserved adjacencies in the matching and **#edg **indicates the number of its edges. X indicates that the exact calculation did not terminate due to the lack of sufficient memory. Click here for file
