# Supplementary material for: Gene family assignment-free comparative genomics
Source: BMC Bioinformatics. 2012 Dec 19;13(Suppl 19):S3. doi: 10.1186/1471-2105-13-S19-S3 (PMC3526435; doi:10.1186/1471-2105-13-S19-S3)
Supplement: Additional file 1 — Measured adjacencies between 12 γ-proteobacterial genomes. Values obtained from FFAdj-Int and FFAdj-MCS. #adj denotes the number of conserved adjacencies in the matching and #edg indicates the number of its edges. X indicates that the exact calculation did not terminate due to the lack of sufficient memory. [file 1471-2105-13-S19-S3-S1.PDF]

A Measured adjacencies between 12 γ-proteobacterial genomes

Values obtained from `FFAdj-Int` and `FFAdj-MCS`. #`adj` denotes the number of conserved adjacencies in the matching and #`edg` indicates the number of its edges. X indicates that the exact calculation did not terminate due to the lack of sufficient memory.

	$\alpha = 0.001$						$\alpha = 0.5$						$\alpha = 1$					
	Exact			Heuristic			Exact			Heuristic			Exact			Heuristic		
Name	$\mathcal{F}_\alpha$	#adj	#edg	$\mathcal{F}_\alpha$	#adj	#edg	$\mathcal{F}_\alpha$	#adj	#edg	$\mathcal{F}_\alpha$	#adj	#edg	$\mathcal{F}_\alpha$	#adj	#edg	$\mathcal{F}_\alpha$	#adj	#edg
BAPHI-ECOLI	201.5	385	559	181.1	401	559	170.6	397	556	128	402	559	140.3	397	550	98.5	402	559
BAPHI-HAEIN	135.8	163	469	134.9	164	469	95	166	467	94	165	469	54.4	169	429	53.1	165	469
BAPHI-PAERU	114.7	233	529	113.3	252	529	86.8	252	527	84.1	253	529	59.5	258	509	57.4	254	529
BAPHI-PMULT	143	191	490	143	191	490	103.5	197	489	103	193	490	64.4	199	446	63.4	194	490
BAPHI-SALTY	201.2	388	559	157.2	401	559	170.3	399	554	128.9	400	559	140.5	398	548	91.6	403	559
BAPHI-WGLOS	111.7	218	423	107.9	225	419	87	225	416	85.1	222	419	62.7	222	357	60	222	419
BAPHI-XAXON	85.5	202	499	83.4	205	499	62.5	204	498	61.8	203	499	39.7	205	461	38.7	205	499
BAPHI-XCAMP	85.2	201	495	82.9	205	495	62.2	202	494	61.3	203	495	39.2	205	456	38.4	204	495
BAPHI-XFAST	81.1	172	467	80.1	173	467	57.9	174	466	57.8	172	467	34.9	176	445	34.5	172	467
BAPHI-YPEST-CO92	197.4	377	558	187.5	382	558	165	379	556	150.3	383	558	133.3	380	548	115.8	385	558
BAPHI-YPEST-KIM	193.8	370	553	164.9	376	553	161.3	373	551	127.1	378	553	128.8	373	543	91.3	380	553
ECOLI-HAEIN	457.2	537	1370	454.3	548	1369	332	566	1357	325.5	558	1370	209.7	573	1265	199.4	561	1370
ECOLI-PAERU	X	X	X	488.5	724	2625	346.8	809	2563	332.9	744	2624	X	X	X	182.6	772	2625
ECOLI-PMULT	521.9	666	1638	515.2	681	1632	390.3	718	1608	380	693	1631	263.5	731	1488	245.5	695	1631
ECOLI-SALTY	X	X	X	2302.5	2833	3411	X	X	X	2080.8	2843	3411	X	X	X	1957.3	2850	3412
ECOLI-WGLOS	170.3	406	607	164.2	414	607	139.3	409	604	131.8	416	607	108.6	407	594	102.7	416	607
ECOLI-XAXON	X	X	X	299.3	517	2084	203.3	552	2063	199.8	531	2084	X	X	X	101.3	551	2088
ECOLI-XCAMP	X	X	X	300.5	506	2075	203.2	540	2052	200.1	519	2073	110.2	606	1667	100.2	541	2073
ECOLI-XFAST	202.3	358	1276	201	380	1267	140.2	403	1259	138.2	390	1267	80.2	427	1102	75.6	396	1270
ECOLI-YPEST-CO92	X	X	X	1271	1725	2668	1148.9	1843	2529	1091.4	1737	2668	X	X	X	931.9	1755	2668
ECOLI-YPEST-KIM	1268.3	1698	2683	1251	1742	2678	1134.1	1850	2546	1065.4	1757	2679	X	X	X	914.5	1773	2680
HAEIN-PAERU	247.5	333	1246	242	377	1246	168.5	385	1234	163.3	384	1248	92.4	411	1139	84.1	385	1247
HAEIN-PMULT	773.2	830	1414	766.2	839	1414	622.1	852	1400	614.8	839	1414	473	853	1303	456.2	841	1414
HAEIN-SALTY	453.9	533	1385	449.8	552	1385	329.2	570	1371	324.7	559	1385	208	576	1268	197.1	562	1385
HAEIN-WGLOS	114.2	174	488	114	175	488	78.8	178	487	78.4	180	488	43.5	177	470	43.1	179	488
HAEIN-XAXON	168	253	1091	166.7	265	1087	110.6	267	1079	108.9	268	1086	54.1	287	944	51.3	276	1085
HAEIN-XCAMP	167.4	251	1078	165.8	264	1070	110.3	267	1065	108.3	266	1070	54.1	283	928	51	272	1070
HAEIN-XFAST	142.1	222	888	141.7	228	887	93.5	234	882	92.8	229	888	44.9	235	710	43.9	231	888
HAEIN-YPEST-CO92	X	X	X	438.1	541	1335	318.8	559	1320	316.3	547	1337	199.1	562	1223	192.2	552	1337
HAEIN-YPEST-KIM	434.4	527	1333	430.7	534	1331	313.6	552	1318	311	540	1333	194.7	556	1211	187.6	543	1334
PAERU-PMULT	279.4	385	1478	277.7	425	1473	192.8	459	1439	188.8	438	1471	X	X	X	99.2	445	1472
PAERU-SALTY	X	X	X	488.9	726	2661	345.7	812	2602	334.4	751	2663	X	X	X	182.4	782	2665
PAERU-WGLOS	104.3	272	573	99.7	284	573	77.4	284	570	72.6	286	573	51.5	290	557	47.2	284	573
PAERU-XAXON	X	X	X	415.1	750	2671	293.7	808	2630	285.2	775	2675	X	X	X	155.8	809	2672
PAERU-XCAMP	X	X	X	413.1	725	2643	290.8	775	2598	282.8	735	2644	X	X	X	153	767	2646
PAERU-XFAST	X	X	X	242.3	490	1392	X	X	X	171.3	496	1391	X	X	X	100.4	505	1391
PAERU-YPEST-CO92	X	X	X	455.1	769	2370	X	X	X	323.4	790	2369	X	X	X	191.9	819	2370
PAERU-YPEST-KIM	X	X	X	446.4	762	2382	327.9	829	2329	315.9	784	2380	X	X	X	185.7	815	2381
PMULT-SALTY	522.7	681	1650	514.9	696	1643	392.3	733	1611	379.3	707	1642	266.6	744	1499	245.4	707	1644
PMULT-WGLOS	121.8	206	520	121.8	208	520	87.5	214	517	87.3	213	520	53.5	215	479	52.7	213	520
PMULT-XAXON	185.4	295	1265	184	314	1253	123.3	328	1251	121.1	321	1250	62.9	352	1075	58.4	327	1249
PMULT-XCAMP	184.9	286	1243	183.4	299	1237	122.7	315	1230	120.6	310	1237	62.3	342	1070	58	316	1237
PMULT-XFAST	154.8	259	972	154.5	264	969	103.8	273	967	103.1	269	969	53.2	282	785	51.6	274	969
PMULT-YPEST-CO92	506.5	658	1600	501.1	680	1594	379.6	714	1574	367.8	690	1594	256.1	724	1433	238.8	692	1594
PMULT-YPEST-KIM	X	X	X	494.1	675	1589	373.1	709	1572	360.8	688	1588	249.4	719	1440	231.4	691	1588
SALTY-WGLOS	169.4	405	606	159.1	412	606	138.7	409	602	126.1	418	606	108.5	407	594	100.4	414	606
SALTY-XAXON	X	X	X	295.3	502	2082	201.7	546	2049	196.7	515	2082	111	607	1698	98.4	534	2086
SALTY-XCAMP	X	X	X	297.7	499	2075	203.6	547	2036	197.4	519	2076	112.1	612	1659	98.2	541	2075
SALTY-XFAST	202.5	370	1300	201.1	394	1291	140.8	420	1279	138.1	403	1291	81.4	448	1124	75.4	412	1291
SALTY-YPEST-CO92	X	X	X	1251.4	1710	2688	1139.5	1831	2559	1062	1727	2691	X	X	X	897	1736	2691
SALTY-YPEST-KIM	X	X	X	1216.8	1718	2705	1126.3	1847	2564	1029.7	1738	2707	X	X	X	864.5	1750	2709
WGLOS-XAXON	81.1	212	537	78.3	217	537	58.1	217	533	55.4	217	537	35.5	223	498	33.1	218	537
WGLOS-XCAMP	81.3	215	539	78.9	219	539	58.3	220	535	56	221	539	35.5	223	503	32.2	221	539
WGLOS-XFAST	75.9	175	490	75.9	175	490	53.3	178	487	53.1	176	490	31.1	179	471	30.4	176	490
WGLOS-YPEST-CO92	169.1	404	604	165.3	409	604	139	405	597	132.8	411	604	109.6	405	589	101.7	412	604
WGLOS-YPEST-KIM	166.1	396	598	160.7	403	598	135.4	395	593	132.6	404	598	105.4	394	584	103.6	401	598
XAXON-XCAMP	2649.6	3197	3641	2228.4	3232	3646	X	X	X	2066.7	3242	3645	X	X	X	1996.1	3250	3644
XAXON-XFAST	X	X	X	727.1	1089	1605	658.1	1094	1579	635.2	1085	1605	555.1	1094	1532	526.7	1087	1604
XAXON-YPEST-CO92	X	X	X	268	497	1888	186	527	1856	180.1	506	1888	105.7	575	1527	92.9	523	1889
XAXON-YPEST-KIM	X	X	X	265.2	484	1906	183.9	523	1876	177.5	497	1906	X	X	X	89.3	519	1909
XCAMP-XFAST	759.6	1068	1601	733.1	1081	1600	656.5	1090	1565	628.3	1080	1600	555.4	1097	1513	521	1088	1601
XCAMP-YPEST-CO92	X	X	X	266.1	485	1861	184.1	516	1835	176.8	496	1860	103.2	566	1500	89.7	508	1859
XCAMP-YPEST-KIM	X	X	X	263	477	1881	181.7	512	1856	174.3	489	1880	X	X	X	86.9	506	1880
XFAST-YPEST-CO92	193.5	365	1230	182.3	382	1226	134.7	397	1217	133.8	390	1224	77.6	413	1061	74.8	394	1224
XFAST-YPEST-KIM	191.2	359	1225	190	372	1224	132.4	387	1215	131.6	380	1223	75.1	407	1055	72.4	386	1222
YPEST-CO92-YPEST-KIM	X	X	X	2969.5	3560	3632	X	X	X	2935.9	3560	3632	X	X	X	2902.2	3560	3632
